# Complement Activation in Autoimmune Bullous Dermatoses: A Comprehensive Review

**DOI:** 10.3389/fimmu.2019.01477

**Published:** 2019-06-26

**Authors:** Gareth Edwards, Gilles F. H. Diercks, Marc A. J. Seelen, Barbara Horvath, Martijn B. A. van Doorn, Jeffrey Damman

**Affiliations:** ^1^Department of Dermatology, University Medical Center Groningen, Groningen, Netherlands; ^2^Department of Pathology, University Medical Center Groningen, Groningen, Netherlands; ^3^Department of Nephrology, University Medical Center Groningen, Groningen, Netherlands; ^4^Department of Dermatology, Erasmus Medical Center Rotterdam, Rotterdam, Netherlands; ^5^Department of Pathology, Erasmus Medical Center Rotterdam, Rotterdam, Netherlands

**Keywords:** complement, auto-immune bullous dermatosis, bullous pemphigod, epidermolysis bullosa acquisita, mucus membrane pemphigoid, pemphigus, linear IgA bullous dermatoses, dermatitis herpetiformis

## Abstract

Autoimmune bullous dermatoses (AIBD) are characterized by circulating autoantibodies that are either directed against epidermal antigens or deposited as immune complexes in the basement membrane zone (BMZ). The complement system (CS) can be activated by autoantibodies, thereby triggering activation of specific complement pathways. Local complement activation induces a pathogenic inflammatory response that eventually results in the formation of a sub- or intraepidermal blister. Deposition of complement components is routinely used as a diagnostic marker for AIBD. Knowledge from different animal models mimicking AIBD and deposition of complement components in human skin biopsies provides more insight into the role of complement in the pathogenesis of the different AIBD. This review outlines the role of the CS in several AIBD including bullous pemphigoid, epidermolysis bullosa acquisita, mucous membrane pemphigoid (MMP), pemphigus, linear IgA-disease, and dermatitis herpetiformis. We also discuss potential therapeutic approaches targeting key complement components, pathways and pathogenic complement-mediated events.

## Introduction

The development of autoimmune bullous dermatoses (AIBD) is a complex interaction between multiple parts of the innate and adaptive immune system. Bullous pemphigoid (BP) is the most frequently encountered AIBD with an annual incidence estimated between 2.4 and 21.7 new cases per million population in different populations worldwide (1). In AIBD, autoantibodies are either directed against epidermal antigens or deposited as immune complexes in the basement membrane zone (BMZ). Besides deposition of immunoglobulins as part of the adaptive immune system, complement components can be found as part of the innate immune system. Secondary tissue injury and blistering can be initiated by complement-dependent and complement-independent mechanisms. Local complement activation triggers the release of cytokines and chemokines that results in the recruitment of inflammatory cells, including neutrophils and eosinophils. This pathogenic inflammatory response eventually results in the formation of sub- or intra-epidermal blisters that will reveal its unique clinical features of the specific disease.

**Figure 1:**
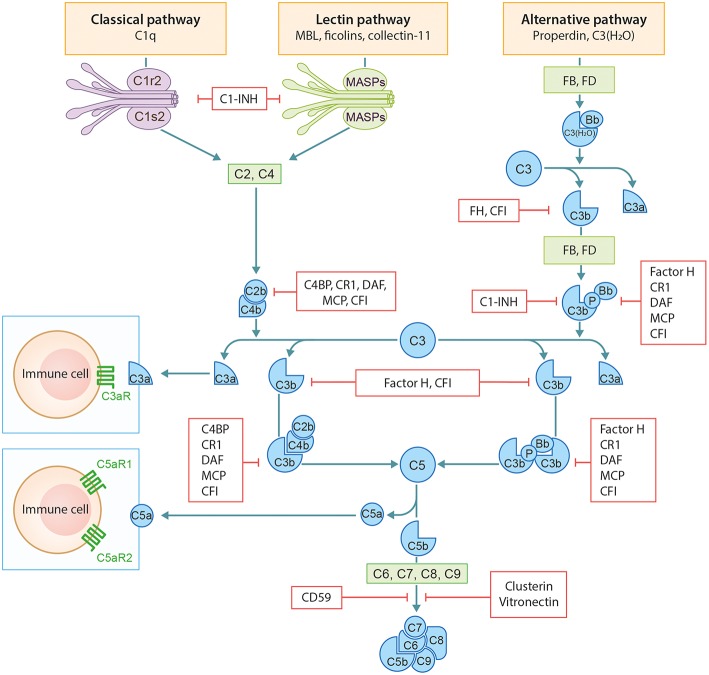
Schematic overview of the complement system. Figure adapted from Giang et al. (2).

The complement system (CS) is an important arm of the innate immune system. It functions as a defense system against pathogens and plays a major role in tissue homeostasis. The CS has three distinct activation routes: the classical pathway (CP), the alternative pathway (AP), and the lectin pathway (LP) (Figure 1). Activation and regulation of the complement system has been extensively outlined before by our group and therefore we refer to our previous publication by Giang et al. for a more detailed description (2).

## Bullous Pemphigoid

**Figure 2:**
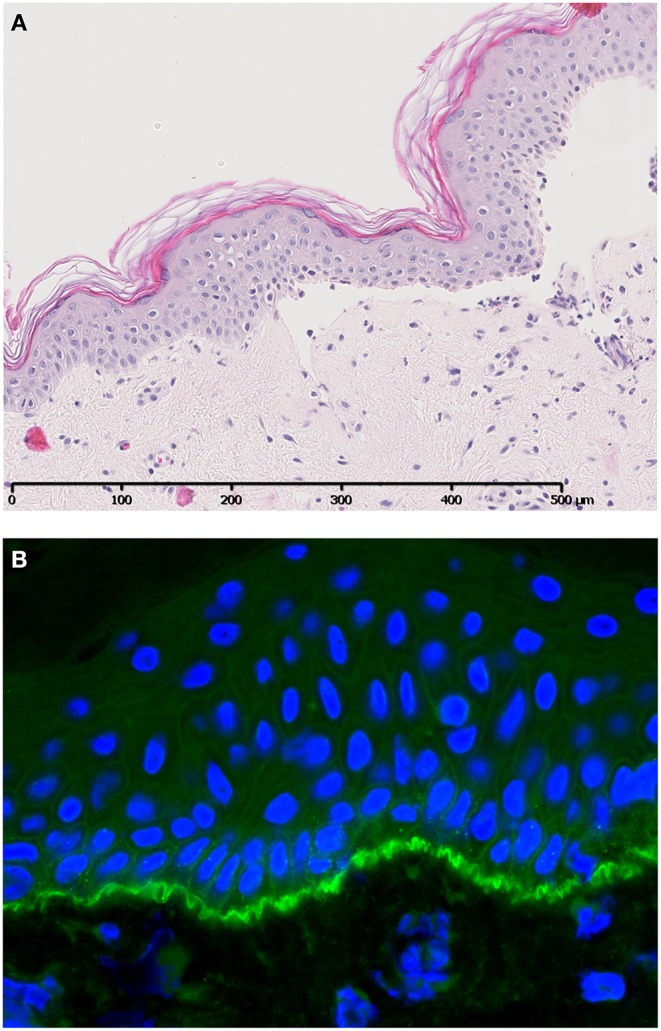
A case of bullous pemphigoid. Histology shows a subepidermal blister **(A)** accompanied by specific linear C3c deposition along the BMZ visualized by direct immunofluorescence **(B)**. Figure (in part) adapted from Giang et al. (2).

Bullous pemphigoid (BP) is an AIBD characterized by autoantibodies against BP180 and/or BP230. Most antibodies in BP are directed against the NC16A domain of BP180, which is a major non-collagenous extracellular antigenic site. Clinically, patients present with tense pruritic dome-shaped, fluid-filled blisters measuring up to several centimeters in diameter. Histology reveals a subepidermal blister that is most often accompanied by an infiltrate of lymphocytes, eosinophils, neutrophils, mast cells, and monocytes/macrophages. Deposition of immunoglobulins and complement factor C3c are routinely used for diagnostic purposes (Figure 2). In addition, deposition of other complement factors of different complement pathways has been demonstrated by direct immunofluorescence (DIF) and immunoperoxidase staining on formalin-fixed paraffin-embedded tissue (3–10). Besides the diagnostic use of complement factors, mouse models have demonstrated an important role for complement in the pathogenesis of BP. In mice, Liu et al. passively transferred a rabbit-antibody against murine BP180NC14A, a homolog of human BP180NC16A. These mice developed a subepidermal blistering disease with a phenotype closely mimicking human BP. Using this model, serum complement depletion by cobra venom factor or C5-deficiency protected against the development of BP. Also, transfer of F(ab′)2-fragments derived from the anti-murine BP180 antibody failed to induce a BP phenotype (11). These findings indicate a crucial role for the IgG-Fc portion and likely the CP, since the FC tail bears the C1q binding region. In line with these findings, C4-KO mice as well as wildtype mice treated with anti-mC1q were resistant to develop BP. Besides a role for the CP, FB-deficient mice developed delayed and less intense blisters, indicating involvement of the AP. It is likely both the CP and AP act in concert when the amplification loop is activated upon CP activation (12, 13). So far, no role has been attributed to activation of the LP. It is questionable whether the previously cited findings can be translated into clinical practice, since BP180NC14a is poorly preserved in mice. For this reasons, humanized mice models were developed replacing mouse BP180NC14A by human BP180NC16A and introducing the human COL17 cDNA transgene into Col17-null mice (14). Importantly, Liu et al. also showed protection against BP in their humanized BP-model after complement depletion with cobra venom factor (15). Also, transfer of humanized IgG1 mutated at the C1q binding site has shown lower pathogenicity in COL17-humanized neonatal mice compared to unmutated humanized IgG1 (16). Although these experiments confirm the complement-dependent blister formation in BP, evidence emerges that also complement-independent mechanisms exist. Karsten et al. have recently demonstrated that the extent of skin lesions decreased with only 50% when C5-deficient mice were injected with anti-COL17 IgG in a humanized mouse model (17). This is in contrast with the previously cited findings in the non-humanized BP-model that showed full protection (11). The differences might be explained by the use of the humanized model by Karsten et al. or the existence of complement-independent mechanisms. Natsuga et al. have demonstrated that rabbit auto-antibodies specifically against the R7 domain within human NC16A induced subepidermal blister formation in COL17-humanized neonatal mice. It was found that the auto-antibody after immunoadsorption against R7, or decoying of R7 with BSA, significantly reduced skin fragility, despite ongoing complement activation. Moreover, F(ab′)2 fragments of anti-BP180 IgG antibodies from BP patients or rabbit IgG against humanized NC16A induced (in part) skin fragility in neonatal humanized mice (18). Lastly, Ujiie et al. found that passive transfer of human BP auto-antibodies induced blister formation in neonatal C3-deficient COL17 humanized mice (19). Altogether, these studies indicate that it is highly likely that also complement independent mechanisms play a role in the pathogenesis of BP. One hypothesis is the direct pathogenic effect of auto-antibodies against NC16A via macropinocytic internalization, ubiquitination and degradation of BP180 leading to impairment of hemidesmosome formation (19, 20). *In vitro* studies indeed demonstrated direct pathogenic effects of auto-antibodies and F(ab′)2 fragments leading to depletion of BP180 in cultured keratinocytes (18, 19, 21). Although complement-independent mechanisms are likely to exist, the contradictory results between different studies could also be explained by the differences in target epitopes on COL17 in the models used, antibody concentration, antibody affinity, or the clonality of antibodies in each experiment (19). Importantly, conflicting results are observed between the humanized and non-humanized models and could (in part) be explained by the lower murine complement fixating and activating capacities of the transferred human auto-antibodies compared to mouse and/or rabbit antibodies (22).

Liu et al. further characterized complement in BP and found that neutrophil depletion protected against the development of BP in wildtype mice, while injection of C5a or IL-8 in C5-KO mice regained susceptibility for BP (23). Moreover, absence of C5aR1-expression on mast cells demonstrated similar protection against BP development, despite complement activation and the presence of neutrophils. It was found that in the BP mouse model, extensive mast cell degranulation occurs that precedes the influx of neutrophil infiltration and subepidermal blister formation. These findings reveal that complement-dependent neutrophil recruitment in BP acts, at least in part, via C5aR1-induced mast cell degranulation (12, 24). Recently, this hypothesis was further investigated by Karsten et al. who confirmed the protection of C5aR1-KO mice against BP, whereas C5aR2-KO animals were more susceptible for disease development. As a proof of principle, prophylactic treatment with a C5aR1-inhibitor PMX53 in mice also led to reduced disease activity (17). Altogether, these results indicate an important role for C5a-C5aR1-axis activation in the development of BP and could be an interesting target for intervention.

Although previous pre-clinical studies are encouraging, these experiments were all performed in mice which makes it difficult to translate these data to humans. However, Romeijn et al. showed in a large patient study comprising 300 patients with BP, that complement C3c was deposited in the majority of (peri)lesional skin biopsies. More interestingly, the extent of deposition was correlated with clinical and serological disease activity, strengthening the crucial role of complement in this disease (25). Besides the deposition of complement in the skin, Chiorean et al. recently found that functional complement-activation capacity of autoantibodies *ex-vivo* in BP also correlates with severity of skin disease and autoantibody titers (26).

## Mucous Membrane Pemphigoid

Mucous membrane pemphigoid (MMP) is an AIBD. The disease represents a spectrum, which affects both skin and mucosa, especially the oral and ocular mucous membranes. Cutaneous lesions manifest as tense blisters that, when ruptured, heal slowly and usually result in scarring. Histopathology reveals a subepidermal blister and a lymphocytic infiltrate in the upper dermis with eosinophils and sometimes neutrophils. DIF shows IgG- and C3c-deposition along the BMZ, but also IgA-, IgM-, and fibrin-deposition can be found (27, 28). Various autoantigens have been found in MMP including BP180 (BPAG2), BP230 (BPAG1), laminin 332, α6/β4 integrin, and laminin 6 (29). Early DIF studies have found deposition of C3, C1q, C4, FB, and properdin along the BMZ (28).

Experimental models that passively transferred rabbit anti-laminin 5 (laminin 332) produced subepidermal blisters with the same clinical, histologic, and immunopathologic features of MMP. However, blistering could not be prevented in C5-deficient mice, indicating that the terminal complement pathway is not involved in blister formation in this experimental model of MMP (30). Also, mice injected with F(ab′)2-fragments instead of intact IgG, developed subepidermal blistering and a clinical MMP phenotype comparable to wildtype mice. These results strongly argue against a complement-dependent mechanism of blister formation in MMP (31). In a recent study by Heppe et al. a mouse MMP model was developed by passive transfer of rabbit anti-laminin 332 specifically against 2 immunodominant regions of LAMα3 in adult mice. The model reflects major clinical and immunopathological characteristics of the human disease. In contrast to the previously described model by Lazarova et al. mice in this model also developed conjunctival lesions and therefore might reflect the clinical situation more accurately. Importantly, clear differences were observed between the two models concerning complement activation. In contrast to Lazarova et al. development of MMP in the model of Heppe et al. was complement-dependent. Mice deficient in C5aR1 or FCqR showed reduced or no signs of MMP. Differences could be explained by the use of IgG against LAMa3 and the use of adult mice by Heppe et al. compared to the use of IgG against all subunits of human laminin 332 and neonatal mice by Lazarova et al. (29, 31).

## Epidermolysis Bullosa Acquisita

Epidermolysis bullosa acquisita is a rare chronic subepidermal AIBD affecting skin and/or mucous membranes. It usually manifests in adults with lesions resembling dystrophic epidermolysis bullosa and is diagnosed after exclusion of all other acquired bullous dermatoses. The disease is characterized by tissue-bound and circulating IgG-autoantibodies against type VII collagen as well as linear u-serrated deposition of complement components along the BMZ (32, 33). In the mechanobullous variant, light microscopy reveals a subepidermal blister, a cell-free subepidermal vesicle, and usually no dermal inflammation. The inflammatory variant frequently resembles histological features of BP.

Experimental mouse models have demonstrated a subepidermal blistering phenotype upon passive administration of rabbit-anti-mouse collagen VII. Moreover, passive transfer of human epidermolysis bullosa acquisita-autoantibodies caused subepidermal blistering and induction of epidermolysis bullosa acquisita skin lesions in mice, proving the pathogenicity of these autoantibodies (34). Similar results were found in an *active* model of epidermolysis bullosa acquisita in which mice developed subepidermal blisters after immunization with collagen VII (35). These mice showed deposition of IgG as well as complement C3 along the BMZ. Comparable to BP, the findings in experimental epidermolysis bullosa acquisita models were also C5 and rabbit IgG-Fc portion dependent and suggests a role for the CP via C1q-FC-receptor binding (34, 36, 37). However, C1q-KO and MBL-KO mice were still susceptible for epidermolysis bullosa acquisita development, comparable to wildtype mice. To further unravel the complement pathways involved, Mihai et al. demonstrated that FB-deficient or wildtype-mice treated with anti-FB developed a delayed and less severe blistering disease compared to wildtype-mice (37, 38). These results attribute an important role to AP activation in the pathogenesis of epidermolysis bullosa acquisita (38). It was hypothesized that the AP serves as an amplification loop upon CP or LP activation, which are likely to compensate for each other. Notably, a role for ficolins in complement activation and amplification loop initiation cannot be fully excluded. Alternatively, AP might be activated upon binding of autoantibodies against complement regulator proteins.

More downstream, epidermolysis bullosa acquisita is likely to be C5aR1-dependent, since C5aR1-deficient animals were protected against development of epidermolysis bullosa acquisita (37, 39). Moreover, animals pretreated with anti-C5aR1 antibody developed a less severe blistering phenotype compared to controls. No protection was found in C6-deficient animals, excluding a major role for MAC formation in epidermolysis bullosa acquisita. In summary, these experimental models demonstrate involvement of CP- and AP-activation and C5a-C5aR1-axis activation in the development of epidermolysis bullosa acquisita.

## Linear IgA Disease

Linear IgA disease (LAD) is defined as a rare group of subepidermal bullous diseases where IgA-autoantibodies target the epidermal BMZ. Patients present with pruritic blisters consisting of grouped vesicles on erythematous bases. The most common antigen targets are LAD-1 and LABD97, which are cleavage products of the extracellular domain of BP180. Histologically, a subepidermal blister is seen with neutrophils aligned along the dermal-epidermal junction or filling up the dermal papillae. DIF shows linear deposition of IgA along the BMZ.

The pathogenesis of LAD can be complement-dependent and -independent. Recent studies have shown that crosslinking of FcαRI on neutrophils by IgA-immune-complexes can directly initiate immune responses and activation and migration of neutrophils in LAD, independent of complement (40, 41). Although IgA is a weak activator of complement in comparison to IgM and IgG, in practice, deposition of C3 can be found in LAD up to 30% (42, 43). Since IgA does not have a C1q-binding site, it cannot activate the CP, but it can activate both the AP and LP (44, 45). It is known from patients with IgA-vasculitis and IgA-nephropathy that particularly the LP can be activated upon binding of MBL and ficolins to IgA deposits in vessel walls or glomeruli (45–47) Whether AP and LP are also involved in the pathogenesis of LAD remains to be investigated. Although complement is deposited in LAD, further research is needed to determine the contribution of complement to the pathogenesis of LAD.

## Dermatitis Herpetiformis

Dermatitis herpetiformis (DH) is a rare cutaneous manifestation of celiac disease that mostly manifests in adults. Patients with DH present with highly pruritic papulovesicular eruptions on various extensor surfaces. Celiac disease is characterized by IgA-autoantibodies against tissue transglutaminase while DH also shows antibodies against epidermal transglutaminase. The tissue transglutaminase modifies a fraction of gluten, gliadin, to an antigen which binds to an HLA-DQ2 or HLA-DQ8 molecule, provoking an autoimmune reaction. The IgA autoantibodies against epidermal transglutaminase are deposited as granular immune-complexes in the dermis. Histology shows characteristic micro-abscesses and granular IgA-deposits in papillary dermis while C3-deposition can be found in up to 89% (48). Seah et al. suggested that complement is activated via the AP since no deposits of C1q were found in DH biopsies (48). Although IgA does not activate the CP of complement, the mere absence of C1q does not exclude CP activation since C1q has a relatively low half-life in tissue. For definitive proof of AP activation, Provost et al. demonstrated specific deposition of FB and properdin as markers of AP activation in DH (49). Also, the role of the LP cannot be ruled out and remains to be investigated. In addition to granular C3-deposition in DH, Preisz and colleagues also found vascular immune precipitates and C3-deposition in DH that strongly co-localized with epidermal transglutaminase (50). As previously discussed in the segment about LAD, the immune reaction in DH can also be complement-independent due to crosslinking of FcαRI with IgA-immune-complexes. However, since the percentage of C3-deposition is higher in DH, this likely to occur less often than in LAD.

## Pemphigus

Pemphigus includes a group of rare chronic blistering diseases characterized by IgG-autoantibodies directed against a variety of desmosomal transmembrane glycoproteins. Patients with pemphigus vulgaris typically present with lesions of the oral mucosa followed by skin-involvement and autoantibodies are directed against epithelial adhesion protein desmoglein 3 and/or desmoglein 1. In pemphigus foliaceus the lesions are localized on the skin, without involvement of the mucous membranes, and autoantibodies are directed against desmoglein 1. DIF studies demonstrate intercellular deposition of IgG and C3c, which suggests involvement of complement activation in the pathogenesis of pemphigus. Furthermore, components of all complement activation pathways can be found in the intercellular substance (ICS) including C1q, properdin, C3, C5, MBL, ficolins, and the MAC (51–53). Nevertheless, animal models have shown that blister formation in pemphigus can be independent of complement activation. In an experimental passive IgG-transfer mouse model, pathogenic patient pemphigus vulgaris IgG and F(ab′)2 were equally pathogenic, while the latter failed to induce complement C3-deposition. In the same model using only pemphigus vulgaris IgG, C5-deficient, or cobra venom factor pretreated neonatal mice also developed pemphigus blisters (54). Although this data indicates a minor role for complement in pemphigus, the results should be interpreted with caution. In contrast to high-dose pemphigus vulgaris IgG, complement-depleted animals transferred with low-dose pemphigus vulgaris IgG showed more limited and delayed onset of disease. Thus, complement activation might not be essential, but potentiates the pathogenicity of autoantibodies in experimental pemphigus. Whether complement plays a role in the initiation and or propagation of pemphigus in humans remains to be investigated.

## Targeting the Complement System in Autoimmune Bullous Dermatoses

The greatest challenge in targeting complement activation, is to find the optimal balance between sufficient blocking and preservation of complement activity. Complete blocking of complement activation is undesirable since this is highly associated with infections. At this moment, no complement targeting drugs have been approved for clinical use in AIBD.

Recently, Kasprick et al. found that treatment with a mouse monoclonal anti-C1s antibody (TNT003) significantly reduced C3c-deposition and production of C4a and C5a, as well as neutrophil chemoattraction in an *ex-vivo* human skin cryosection assay (55). These results show that early inhibition of the CP can prevent IgG-induced complement activation *in-vitro*. Although the *in vivo* effect on inflammation or blistering is unknown, this data provides rationale to test Sutimlimab, a humanized monoclonal of TNT003, which was shown to be safe and well-tolerated in humans (56, 57). Selective CP inhibition without interfering in AP and LP opens new and safe opportunities for interventions in BP, thereby leaving important arms of the CS intact.

Other possible ways to inhibit the CS includes the inhibition of the C5a-C5aR axis. The anti-C5 monoclonal antibody Eculizumab binds to C5, thereby preventing cleavage into its active components, but has been associated with a thousand-fold increased risk of meningococcal disease (58). Coversin (a recombinant small protein) is another anti-C5 drug that is currently in phase IIA of clinical trials in BP-patients. In addition of binding C5, it also inhibits leukotriene B4, another powerful inflammatory mediator (https://adisinsight.springer.com/trials/700284185). Although C5-blocking drugs are interesting, it will also interfere with C5a-C5aR2-axis activation, which has recently shown protective effects in BP (17). Therefore, inhibition of C5a-C5aR1 axis might be more interesting, leaving C5a-C5aR2 interaction intact. Avacopan is an oral small molecule C5aR1-inhibitor and has shown promising results in clinical trials by having a long half-life and showing limited side effects (59).

Further studies are needed to investigate the mechanisms of complement activation in humans which provides a basis for developing more specific therapeutic approaches targeting key complement component, pathways and pathogenic events.

## Conclusion

It has been shown through various experimental and clinical studies that the CS plays a role in several AIBD. In animals models using complement-deficient mice, different complement pathways are involved in the pathogenesis of different AIBD. Although recent clinical studies in BP found association of complement with disease activity, it still remains to be investigated how important the CS is in the pathogenesis of other AIBD. In the last few years multiple drugs targeting specific parts of the CS are in clinical development and some of these have shown promising results in preclinical studies. Selective neutralization of pathway-specific or terminal cascade specific components is promising, since this leaves most other parts of the CS intact. As with all new drugs, the ratio of risk to benefit for the patient must be assessed in future clinical trials.

## Author Contributions

GE, GD, MvD, and JD: wrote the review. MS and BH: edited and approved the manuscript.

### Conflict of Interest Statement

The authors declare that the research was conducted in the absence of any commercial or financial relationships that could be construed as a potential conflict of interest.

## Abbreviations

AP: alternative pathway
AIBD: autoimmune bullous dermatoses
BMZ: basement membrane zone
BP: bullous pemphigoid
C5aR1: C5a receptor 1
C5aR2: C5a receptor 2
CP: classical pathway
CS: complement system
DH: dermatitis herpetiformis
DIF: direct immunofluorescence
KO: knock-out
LAD: Linear IgA disease
LP: lectin pathway
MAC: membrane attack complex
MBL: mannan binding lectin
MMP: mucous membrane pemphigoid.
